# Selenium and Sulfur to Produce *Allium* Functional Crops

**DOI:** 10.3390/molecules22040558

**Published:** 2017-03-30

**Authors:** Susana González-Morales, Fabián Pérez-Labrada, Ema Laura García-Enciso, Paola Leija-Martínez, Julia Medrano-Macías, Irma Esther Dávila-Rangel, Antonio Juárez-Maldonado, Erika Nohemí Rivas-Martínez, Adalberto Benavides-Mendoza

**Affiliations:** 1 CONACYT-Universidad Autónoma Agraria Antonio Narro, Saltillo 25315, Mexico; sgonzalezmo@conacyt.mx; 2 Departamento de Horticultura, Universidad Autónoma Agraria Antonio Narro, Saltillo 25315, Mexico; fabperlab@outlook.com (F.P.-L.); emlaugaren@gmail.com (E.L.G.-E.); pclm15@hotmail.com (P.L.-M.); dari_86@hotmail.com (I.E.D.-R.); erikanohemi257@gmail.com (E.N.R.-M.); 3 Facultad de Ciencias Biológicas, Universidad Autónoma de Nuevo León, Ave. Pedro de Alba s/n, Ciudad Universitaria, San Nicolás de los Garza 66450, Mexico; jmedmac@gmail.com; 4 Departamento de Botánica, Universidad Autónoma Agraria Antonio Narro, Saltillo 25315, Mexico; juma841025@hotmail.com

**Keywords:** nutritional quality, biofortification, phytochemicals of *Allium*, sulfur metabolism, selenium metabolism

## Abstract

Selenium is an element that must be considered in the nutrition of certain crops since its use allows the obtaining of biofortified crops with a positive impact on human health. The objective of this review is to present the information on the use of Se and S in the cultivation of plants of the genus *Allium*. The main proposal is to use *Allium* as specialist plants for biofortification with Se and S, considering the natural ability to accumulate both elements in different phytochemicals, which promotes the functional value of *Allium*. In spite of this, in the agricultural production of these species, the addition of sulfur is not realized to obtain functional foods and plants more resistant; it is only sought to cover the necessary requirements for growth. On the other hand, selenium does not appear in the agronomic management plans of most of the producers. Including S and Se fertilization as part of agronomic management can substantially improve *Allium* crop production. *Allium* species may be suitable to carry out biofortification with Se; this practice can be combined with the intensive use of S to obtain crops with higher production and sensory, nutritional, and functional quality.

## 1. Introduction

It is known that cultivated terrestrial plants require at least 17 elements for their metabolism, growth, and reproduction [1]. In the case of humans, the essential elements are at least 28 [2]. This difference has the consequence that, in practice, several elements that are important in human nutrition are not considered in crop nutrition programs, especially in the case of crops cultivated in soilless systems. The ideal number of elements to be considered for the nutrition of plants destined for human consumption should be 20, that is, the 17 elements considered essential for plants, in addition to selenium, silicon, and iodine.

However, not all species of crop plants have the same ability to absorb, metabolize, and accumulate these three additional elements. For example, some species of *Brassicaceae*, *Fabaceae*, *Asteraceae*, and *Alliaceae* can absorb and accumulate selenium [3,4,5,6]; *Poaceae*, *Fabaceae* and *Cucurbitaceae* do the same with silicon [7]; and *Laminaria* algae stand out with iodine [8].

Biofortification with one or more of the three elements mentioned would have different results according to the taxonomic group or species of plants in which the process is carried out. Among the groups of plants that may potentially be good alternatives for selenium biofortification are those that by nature accumulate many sulfur compounds in their tissues, such as *Brasicaceae* and those of the genus *Allium*. Climate change is expected to have an adverse effect on selenium availability in agricultural soils [9]. Hence the importance of directing biofortification efforts towards crops like *Allium* that have an exceptional ability to absorb, metabolize and store selenium.

The genus *Allium* includes more than 550 species distributed throughout the world in temperate, tropical, and semi-arid regions. Some species are of great importance for their culinary, medicinal, and ornamental use [10]. Some of the species that stand out are: garlic (*A. sativum*), wild garlic (*A. ursinum*), elephant garlic (*A. ampeloprasum* L. var. *ampeloprasum*), white garlic (*A. neapolitanum*), onion (*A. cepa* L.), chives (*A. fistulosum*), garlic onion or scallion (*A. schoenoprasum* L.), Chinese chives (*A. tuberosum* L.), and leek (*A. ampeloprasum* L. var. *porrum*). All are of great importance for being edible plants and for their use in medicine as antimicrobial, lipid-lowering, hypocholesterolemic, antithrombotic, cardiovascular, hypoglycemic and antitumorigenic [11,12].

A large number of enzymes involved in the metabolism of assimilation and volatilization of S are functional in the presence of Se. The ability to accumulate S and Se in a certain species will depend on their ability to transform ionic forms into more stable organic forms that can be stored and fulfill certain metabolic functions [13]. On the other hand, the concentration of S and Se in plant tissues also depends on the balance between absorption, transport, and assimilation with the volatilization process; such activities occur for both S and Se [14]. Many plant species and accompanying microbiomes volatilize Se when this is highly available [15,16]; similarly, the rate of sulfur volatilization is inversely related to the concentration of sulfate in the growth medium [17]. *Allium* plants do not show such a great volatilization activity in the presence of high concentration of S and Se, allowing to obtain crops enriched in both sulfur and selenium that can be an excellent dietary source of these elements [16].

The objective of this manuscript is to present an overview of the information on the use of Se and S in the cultivation of plants of the genus *Allium*, mentioning the processes of absorption, transport and assimilation, reviewing the forms and types of application of both elements, highlighting the impact on quality and productivity, as well as the concentration of phytochemicals that determine the nutraceutical value of the crops. The idea is, given the characteristics already mentioned, to propose to *Allium* as a suitable model for biofortification with Se and S for human consumption purposes.

## 2. Absorption and Metabolism of Sulfur and Selenium in *Allium*

In every terrestrial plant species, the assimilation of selenium is carried out through the metabolic absorption route of sulfur [18]. However, in species of the genus *Allium*, a greater capacity to absorb, metabolize, and assimilate S and Se have been found. A characteristic that gives *Alliaceae* such capacity is that they can methylate the seleno-amino acids, thus reducing the rate of incorporation of the same in proteins and, on the other hand, increasing, if necessary, the rate of volatilization of Se. That is why the concentration of S and Se and its metabolites in this group of plants is very high compared to other groups [19]. Indeed, if the natural concentration of selenium in wheat grain in the UK (0.0155–0.0438 mg·kg^−1^) [20] and rice in some regions of China (0.015–0.046 mg·kg^−1^) [21] is compared to selenium levels of onion (0.024–0.5 mg·kg^−1^) and garlic (0.015–0.5 mg·kg^−1^) [22,23] cultivated in low selenium soils, the highest levels for *Allium* sp. exceed ten times those of the grasses. When biofortifying the grasses with Se, they reach 1.64 mg·kg^−1^ [24], while the onion shows 28–140 mg·kg^−1^ and the garlic 68–1355 mg·kg^−1^ [22,25]. In biofortified *Allium tricoccum*, the Se level ranges from 48 to 784 mg·kg^−1^ [22,26]. Something similar is observed with sulfur: the ranges of adequate concentration of total sulfur in wheat and maize range from 300 to 8900 mg·kg^−1^ [27], while for garlic are 4600–6000 [28] and for onion are 1540–5350 [29].

### 2.1. Absorption

In soil, selenium can be found as selenide (Se^2−^), elemental Se (Se^0)^, trioxide diselenium (Se_2_O_3_^2−^), or selenate (SeO_4_^2−^) [30,31,32]. Sulfur may be found in soils in the form of sulfide (H_2_S), elemental sulfur (S°), sulfate (SO_4_^2−^), thiosulfate (S_2_O_3_^2−^), tetrathionate (S_4_O_6_^2−^), or as thiols, disulfides, sulfones, and sulfonic acids [33,34,35,36,37]. The behavior of the different chemical species of Se and S as well as their solubility are directly related to the characteristics of the soil, microbial activity, pH, oxidation-reduction potential, and biological methylation processes in the case of Se [30,31,36,37,38,39].

**Figure 1:**
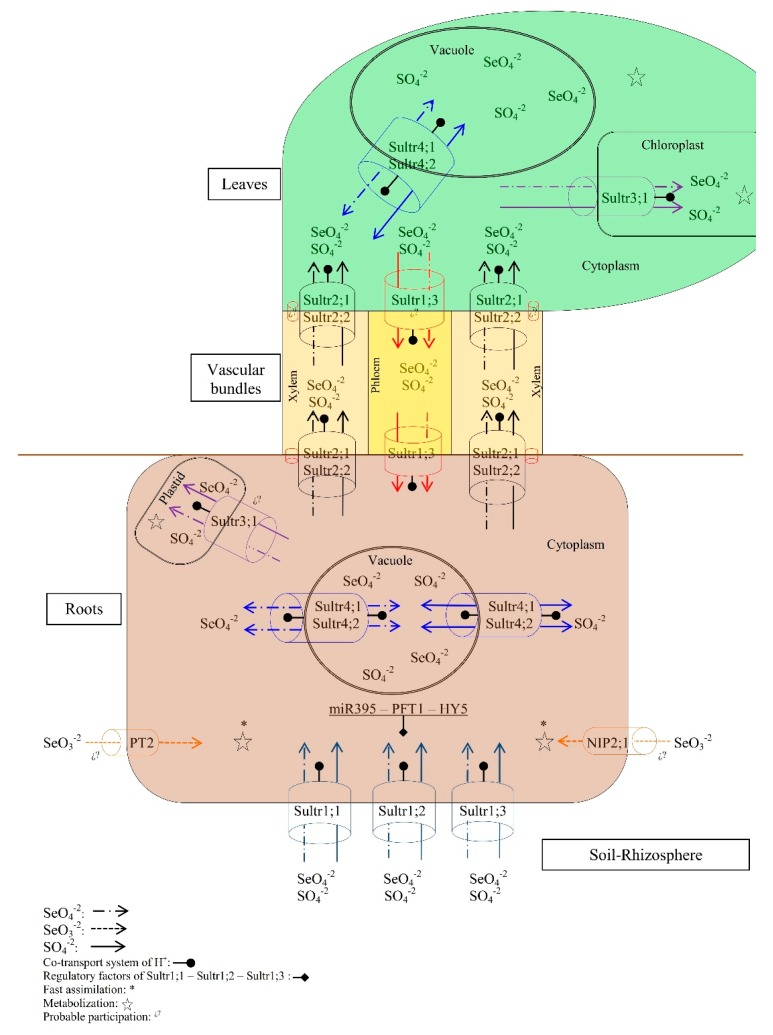
Estimated model of absorption and transport processes of S and Se in *Allium*. The Se and S present in the soil are absorbed in a higher proportion as selenate (SeO_4_^2−^) and sulfate (SO_4_^2−^) by H+ co-transport through the high-affinity sulfate transporters (HAST): Sultr1;1, Sultr1;2 and Sultr1;3 that are present in the plasmatic membrane of the root. Once absorbed, SeO_4_^2−^ and SO_4_^2−^ can be immediately metabolized into the cytoplasm, enter the vacuole using the Sultr4;1 and Sultr4;2 transporters present in the tonoplast membrane to be stored, or mobilized into the plastids (probably by the Sultr3;1 transporter) where they are metabolized, or they can be mobilized to the leaf tissues by the low-affinity sulfate transporters (LATS) Sultr2;1 and Sultr2;2 by loading and unloading the leaf xylem. Such transporters are regulated by Sultr1;3. In the leaf, SeO_4_^2−^ and SO_4_^2−^ can be metabolized in the cytoplasm, enter the vacuole (by the transported Sultr4;1) or the chloroplast (by transporter Sultr3;1) where they are metabolized, or remobilized towards the root via phloem through the Sultr1:3 transporters. Se may also be absorbed as SeO_3_^2−^ to a lesser extent by the phosphate (PT2) and silicon (NIP2;1) transporters, being immediately metabolized in the cytoplasm.

Plants have preferences for certain chemical forms to absorb selenium and sulfur. Selenium is taken from the soil solution mainly in the form of selenate (SeO_4_^2−^) and in less quantity as selenite (SeO_3_^2−^) or organoselenium compounds (selenocysteine and selenomethionine) [38,40,41,42]. On the other hand, the roots incorporate sulfur preferably as sulfate (SO_4_^2−^), since it is a more stable compound in the soil, and to a lesser extent as thiosulfate (S_2_O_3_^2−^) [34,36] (Figure 1).

Taking into account that the Se and S share very similar properties, Se is absorbed, translocated and metabolized by mechanisms analogous to those of S [40]. As far as we know there are no reports of the transporters of selenate or sulfate in *Allium*. However, considering the reported analogy for sulfate transporters in various plant species [43], it can be assumed that the mechanisms described in the *Arabidopsis thaliana* model plant are applicable in *Allium*. In *A. thaliana* selenate is captured through active uptake by sulfate transporters present in root cells [38,40]; such transporters are encoded by at least fourteen genes in *Arabidopsis*. These genes have been classified into five groups according to the characteristics of the coding proteins, all of which are H+/sulfate co-transporters [44,45]. Transporters Sultr1;1, Sultr1;2 and Sultr1;3 are high-affinity sulfate transporters (HAST) which are induced by low sulfate concentration and the presence of selenate [46,47,48]. HAST mutants in arabidopsis show a considerable reduction in selenate uptake [48]. Thus they are considered important for the selenate uptake [13].

It should be noted that the absorption of selenate competes with the uptake of sulfate [42]. The contributions of Se as selenate, induce the decrease in the concentration of S metabolites, even when there are high levels of available sulfate [49]; similarly, it reduces the concentration of S in the bulbs of different onion cultivars by increasing the concentration of selenate applied [50]. This fact indicates the need to ensure the adequate balance of S:Se for *Allium* when selenium biofortification is pursued.

### 2.2. Transport

HAST are accompanied at the epidermis, cortex, and parenchyma by low-affinity sulfate transporters (LAST) which function cooperatively with HAST, with the difference that LAST has a lower response to selenate. It has been found a greater relative abundance of HAST in the epidermis and cortex of the root, whereas the LAST appear with greater profusion in the parenchyma associated to the xylem and phloem [51,52,53]. Once the sulfate is placed in the radical cells, one part is stored in the vacuoles and redistributed later using the Sultr4;1 and Sultr4;2 transporters of the tonoplast [54]. Another part of the sulfate is translocated to the xylem, and thence to stems and leaves, by the low-affinity transporters Sultr2;1, Sultr2;2 [52] and by the high-affinity Sultr1;3 [55].

**Figure 2:**
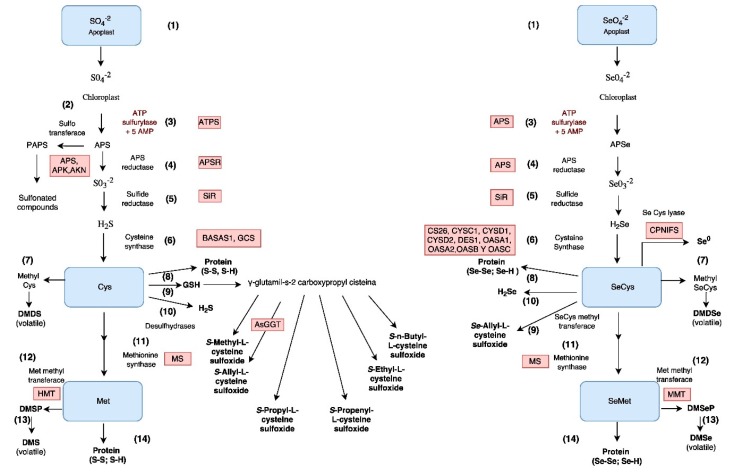
Comparing the assimilation pathways of: sulfur (**left**); and selenium (**right**), in plants of the genus *Allium*. After absorption of the sulfate and the selenate in the roots, these are transported to the leaves via apoplast (1); in the plastids are reduced to sulfide and selenide respectively. The first step is the activation of the molecule 5′AMP, by the action of the enzyme ATP Sulfurylase (3), encoded in *Allium* by the ATPS gene in the S pathway, and by the APS gene and isoforms in the Se pathway, forming 5′-adenosinphosphosulfate (APS) and APSe respectively. From these compounds arise the so-called primary route and secondary route or pathway of sulfation. In the primary route, APS (4) is reduced to sulfite (SO_3_^2−^) and APSe to selenite (SeO_3_^2−^) by APS reductase, which is encoded by the APSR gene in the S pathway and by the APS gene in the Se route. Subsequently, the sulfite and selenite are converted to sulfide and selenide, by the action of sulfite reductase, encoded by the SiR gene in the path of S. The gene encoding this enzyme in the Se pathway is not yet known. Subsequently, sulfide and selenide will be incorporated into the skeleton of acetyl serine by the enzymatic complex cysteine synthase (6), formed by the acetyl serine transferase and O-acetylserinatiolase, resulting in the formation of cysteine or selenocysteine. In *Allium*, the genes encoding this enzymatic complex are BASAS1 for the S and GCS pathway, along with their isoforms. For the Se path, it is thought to be similar to that reported in arabidopsis, where the genes CS26, CYSC1, CYSD1, CYSD2, DES1, OASA1, OASA2, OASB, and OASC participate. The amino acids cysteine and selenocysteine are the starting point of various metabolic pathways such as the formation of dimethyldisulfide by the methylation of Cys or dimethyl diselenide by the methylation of SeCys (7), incorporation into proteins (8) and formation of glutathione (GSH) in the S pathway and the sulfoxide Se-allyl-l-cysteine in Se (9). From the GSH the sulfoxides are synthesized, which originate thiosulfinates, volatile compounds characteristic of *Allium*, as well as the formation of hydrogen sulfide by the enzyme desulfhydrase and seleniuric acid (10), or the synthesis of the amino acid methionine, or its equivalent, selenium methionine, by the action of the enzyme methionine synthase, encoded for both elements by the MS gene in arabidopsis (11). From methionine, dimethylsulfide propionate (DMSP) or DMSeP can be obtained using the enzyme methionine methyltransferase, which is encoded by the HMT gene for S and by the MMT gene for Se, hence the synthesis of dimethyl sulfide DMS or DMSe which are volatile compounds (13). In the secondary or sulfation route, phosphoadenosine phosphosulfate (PAPS) is synthesized from APS, catalyzed by the enzyme sulfotransferase (2), resulting in sulfonated compounds such as glucosinolates (2). This secondary route has not been observed for Se.

According to the internal demand, another part of the absorbed sulfate can immediately be assimilated into the radical tissues through the primary route (Figure 2) [56]. In the root pith parenchyma, sulfate (like selenate) is loaded by the LAST (Sultr2;1 and Sultr2;2) into the xylem and the dynamics of its transport and distribution partially follow the flow of transpiration, being able to regulate the load of sulfate to the xylem (influx/efflux) in the radical cells, even in wide ranges of sulfate concentration (10.4–20.8 mM) [57]. The distribution to other plant organs appears to heavily depend on events associated with development [58], which indicates the importance of adequate nutrition with N, P, K, Ca, and S as well as the consideration of external environmental factors such as temperature and irradiance that determine the rate of growth [59], or internal factors as growth regulators [60,61], since these can be determinants in the accumulation of S and Se in cultivated *Allium*.

From the xylem, the sulfate is discharged into the apoplast, or via the symplastic pathway in the cytoplasm of the leaf cells by HAST and LAST Sultr1;3, Sultr2;1 and Sultr2;2. A fraction of the sulfate is stored in the vacuole, and the rest is mobilized by Sultr3;1 to the chloroplasts where it is assimilated into organic forms [62,63] (Figure 2). If necessary, leaf sulfate is remobilized from the vacuole by Sultr4;1 [54]. Although plastids from non-photosynthetic tissues have the necessary metabolic mechanisms for sulfate reduction and assimilation, this process of sulfate assimilation occurs mainly in photosynthetic tissues [45].

The Se transport pathway appears to be very similar to that described for sulfate, even using the same carriers previously mentioned [38]. The storage of selenium in ionic form has not been confirmed in the vacuole, however selenium appears in significant concentration in inorganic form in *A. fistulosum* when 100 mg·L^−1^ of selenite or SeMet is applied, which may indicate a storage of inorganic Se in apoplast or vacuole or both, in cases of high availability of selenium [41].

In onion the transport mechanisms of Se differ as a function of whether the element is absorbed as selenate or selenite [64]. Selenate is absorbed and moves via apoplast, which seems to reduce the toxicity of selenate for the radical cells of onion. To transport selenate to other organs of the plant, one part appears to be reduced to Se^2−^, which allows absorbing a large amount of selenium without intoxication. On the other hand, when onion is exposed to selenite, transport seems to occur in a symplastic way, since absorbed SeO_3_^2−^ is immediately metabolized to form Se-cysteine and Se-methionine [42,64]. If available in the soil, certain organic forms of Se (such as selenomethionine) may be actively absorbed by the roots [42]. As in other species, in *A. ampeloprasum*, the selenate uptake is much more effective than selenite uptake [65].

### 2.3. Assimilation

Once in the leaf cells, both selenate and sulfate are assimilated in a similar way [13,49] (Figure 2). Both compounds are initially activated by reacting with ATP in the presence of the ATP sulfurylase enzyme to form APS (adenosine-5-phosphosulfate) or APSe (adenosine-5-phosphoselenate) [66]; subsequently, a reduction process is carried out to selenide (Se^2−^) or sulfide (S^2−^) [45], giving rise to the organic incorporation that can take two paths, the first consists of the synthesis of the amino acid cysteine (Cys) or Se cysteine (Secys), and is called the primary path which has been described for both Se and S [67]. The second pathway is carried out for the synthesis of secondary metabolites, resulting in the synthesis of PAPS (3-phosphoadenosine-5-phosphosulfate) from APS, has been confirmed only for sulfur, and is called secondary or sulfation path. In the secondary pathway, a series of sulfotransferases transfer sulfonate (RSO_3_^−^) groups in the cytoplasm to a wide range of substrates and allow the synthesis of glucosinolates and other compounds. The absence of a secondary assimilation pathway for selenate is possibly related to the absence of selenate esters in plants, and the fact that selenoamino acids, such as selenium-methyl selenocysteine, are the most common products of the assimilation of Se in the species of the genus *Allium* [13].

In the primary pathway, following APS or APSe synthesis, these compounds are reduced to sulfite (SO_3_^2−^) or selenite (SeO_3_^2−^) (Figure 2) by the enzyme APS reductase [66,68]. Subsequently, the enzyme sulfite reductase reduces sulfite to sulfide (S^2−^) and selenite to selenide (Se^2−^) [68,69], whose protonated forms (H_2_S and H_2_Se) are incorporated into the skeleton of acetyl serine by the cysteine synthase complex (serine acetyltransferase + *O*-acetyl serine (thiol) lyase) to obtain the amino acids cysteine or selenocysteine [70,71]. These amino acids are the starting point of various metabolic pathways, some similar to both elements, such as the formation of methionine and selenomethionine, cystathionine and homocysteine, protein synthesis and selenoproteins, as well as different volatile molecules. On the other hand, from the sulfur amino acids are obtained another series of compounds like methiin (C_4_H_9_NO_3_S, present in most alliums), alliin (C_6_H_11_NO_3_S, characteristic of garlic), isoalliin (characteristic of onion) and propiin (C_6_H_13_NO_3_S, present on the onion) [72,73]. According to Jones et al. [72], the chemical intermediates precursors of alliin in *Allium* are compartmentalized in different organelles according to the species; for example, alliinase is observed in the vacuoles of all cells in onion, whereas in garlic it is found only in the vacuole of the cells of the vascular bundle sheath.

Methylation of cysteine and selenocysteine produce methyl cysteine and methyl selenocysteine, which, through oxygenation of sulfur, form compounds called methyl cysteine sulfoxide (DMDS) and methyl selenocysteine sulfoxide (DMDSe), respectively. In *Allium* and in *Brassicacea*, these compounds, apparently used as defense against biotic stress, are metabolized after mechanical damage by the cysteine sulfoxide lyases, producing methanesulfonic acid, which in turn is transformed into another series of compounds of possible defensive value such as methanethiol and dimethyl disulfide sulfoxide [74]. 

On the other hand, the methylation of methionine, and its selenoequivalent produces compounds called dimethylsulfoniopropionate (DMSP) and dimethylsulfonioselenate (DMSeP). Although both compounds may be metabolized to the volatile compounds dimethylsulfide (DMS) and dimethylselenide (DMSe), the amount of DMSP that is converted to DMS is very low, whereby these compounds are believed to fulfill storage functions, osmoregulation, protection against herbivores and oxidative damage. Another proposed role for DMSP and DMSeP is as metabolites produced for the removal of the excess (detoxification) of S or Se [75]. The DMS has great ecological importance since when it reaches the atmosphere, it is converted by photo-oxidation to other sulfur compounds including oxyacids and inorganic sulfates which subsequently return to the earth in the form of acid rain; this process has been found in terrestrial plants. However, the greatest contribution comes from the ocean [76]. Such ecological effects are not known for DMSe molecules.

Another mechanism of detoxification of Se is through the formation of elemental selenium (Se^0^) by the breakdown of SeCys through the catalysis of the enzyme SeCys lyase reported in *A. fistulosum* [66,77]. For sulfur, the formation of the elemental form (S^0^) has been reported as a defense against pathogens [78]; its biosynthetic pathway is not fully established but it is probably released from glutathione [79].

The diversity of defense compounds or volatile compounds with selenium appears to be much lower compared to that shown by sulfur compounds, perhaps because of the increased opportunity for sulfur to form chemical variants with the propenyl groups (CH=CHCH_3_) (which form the nucleus of sulfoxides, as the allicin C_6_H_10_OS_2_ characteristic of garlic). In selenium, preference is given to methylated compounds, especially for *Allium* [41], and few natural analogs are known with Se of the sulfuric propenyl compounds, characteristic of *Allium*.

## 3. Phytochemicals of *Allium* spp. Derived of Se and S

In *Allium* spp., the metabolism of sulfur, after cysteine synthesis, differs from other groups of plants, since the synthesis of an extensive battery of sulfur compounds occurs. These compounds are traditionally associated with the scents and flavors of *Alliaceae* [72], but fulfill other functions such as sulfur storage, cellular redox balance, antioxidant protection and stress defense [80,81,82]. In the sulfur route, phytochemicals are mostly represented by glucosinolates, used in defense against different types of stress [83]. In addition, specific secondary routes are used. In the case of sulfur, the formation of a wide range of defense molecules, such as H_2_S and GHS, is included. From these derives the synthesis of the sulfoxides precursors of the volatile molecules that give the smell and characteristic organoleptic properties to the alliaceous. H_2_S is used by the plant as a defense against pathogens [78]. However, it is also considered as part of a mechanism of regulation in the accumulation of cysteine [84]. The functions of glutathione (GSH) are important for the maintenance of redox status in the cell, as an antioxidant and precursor of *S*-alk(en)yl cysteine sulfoxides methiin, alliin, propiin, isoalliin, ethiin, and butiin. These non-protein sulfur amino acids are hydrolyzed by the enzyme alliinase to produce flavor and pungency imparting compounds in *Allium* [81]. Randle and Lancaster [85] reviewed the sulfur’s compounds related with the flavor in *Allium*. Although most of the enzymes involved in the biosynthesis of these compounds have not been identified in *Allium*, the AsGGT1, AsGGT2, and AsGGT3 genes of garlic have been described. These genes encode the enzyme γ-glutamyltranspeptidase (GGTs), which suggest that they may contribute in a different way to the biosynthesis of alliin in garlic [86]. Recent findings have characterized a compound analog to alliin, this is a precursor sulfoxide of allicin derived from selenium metabolism [87].

An alternative in the metabolism of sulfur amino acids leads to the synthesis of volatile compounds, such as H_2_S, or volatile methylated compounds such as DMDS, DMSP, and DMS. These compounds are produced by some living organisms, including anaerobic bacteria [88], seaweed [89] and plants [90], and are widely associated with marine waters, wetlands, decomposition of organic matter, and volcanic emissions [91]. The physiological function of these compounds is mainly associated with sulfur dissipation [92,93] as regulator and signal in the stress response [94,95], and is also involved in biogeochemical processes [96,97,98].

In the selenium route, analogous compounds are synthesized. However, the function and chemical nature of these compounds are not entirely described. Se-methyl selenocysteine (SeMeSeCys) is considered the most abundant Se compound in garlic, onion, and *A. ampeloprasum* when supplemented with Se [4,99]. It is thought that the synthesis of SeMeSeCys is part of a mechanism of tolerance to Se in plants, allowing the conversion of potentially toxic selenoamino acids to non-protein derivatives such as MeSeCys [100,101]. Likewise, compounds such as DMDSe, DMSeP, and DMSe are considered part of a strategy to increase tolerance to Se, by producing volatile forms of Se [102].

### Impact of Se and S on the Nutritional and Functional Quality of *Allium spp.*

The plants that integrate the genus *Allium* have been used since ancient times because of the multiple beneficial effects on human health such as antiasthmatic, hypolipemic, antithrombotic, anticarcinogenic, antimicrobial, and hypoglycemic actions [81]. The most studied phytochemicals of *Allium* are sulfur compounds. As Figure 2 illustrates, some of these compounds may appear as forms containing selenium instead of sulfur. Table 1 presents results on the biofortification of *Allium* with S and Se and the impact on some phytochemicals.

Garlic is the species that has been shown by in vitro and in vivo studies to be the species with the greatest number of beneficial effects on human health, due to its higher concentration of sulfur compounds [103].

The biological activity of the sulfur compounds is linked to the level of unsaturation and asymmetry in the molecules, the cepaenes, a class of structurally related α-sulfinyl disulfides [104], having two double bonds (e.g., bis[2-methyl-1-(1-methylethenyl)-1-propenyl] disulfide) are more active than those having a single, double bond (e.g., methyl (*E*)-1-(1-propenylthio)propyl disulfide), and than thiosulfinates with lower level of unsaturation (e.g., methyl allyl-thiosulfinate). In addition, thiosulfinates with aromatic and poly-substituted substituents (e.g., *S*-phenyl 2,2-dimethyl-propane-thiosulfinate) are more reactive than those lacking these chemical characteristics (e.g., Dimethyl thiosulfinate) [105]. These compounds are usually extracted using organic solvents (methanol, ethanol, etc.) while sulfoxides and some phenolic compounds such as quercetin or other antioxidants are isolated by aqueous extraction. Therefore, the beneficial effects of extracts of *Allium* species depend on the polarity of the extractant in conjunction with the chemical nature of the extracted compounds.

In humans, the organosulfur compounds of *Allium* are associated with the modulation of the activity of enzymes such as glutathione S-transferase (GST), quinone reductase (NQO1), and UGT-glucuronosyltransferase (UGDT), which are important in the detoxification of carcinogenic compounds [81,106,107]. *Allium*’s anticancer and antiproliferative activity, as well as antimicrobial capacity against a broad spectrum of infectious agents, is attributed to the effect of allicin, which is highly permeable through membranes [108], and undergoes a thiol-disulfide exchange reaction with free thiol groups present in the proteins. It is believed that these properties are the basis of its antimicrobial effect [109], having effects against different bacteria, fungi, and yeasts [110].

Similarly, the unsaturated trisulfide compounds (as diallyl trisulfide) have potent anticancer activity, which has been tested in colon adenocarcinoma, prostate, and lung cancer [111,112,113,114]. The mechanisms of action described are the induction of apoptosis [115], inhibition of malignant cell growth in vitro [116] and inhibition of adenomas [117]. Sulfur compounds containing more sulfur atoms mitigate the damage caused by diabetes [118].

Aqueous extracts of garlic and *A. ampeloprasum* have been shown to be effective in reducing *N*-nitrosorpholine (liver carcinogen). *A. ampeloprasum* is also effective against several types of malignant cells inducing apoptosis and necrosis [119].

*A. ampeloprasum* has several antioxidant, anticancer, antimicrobial, hepatoprotective, antidiabetic, anti-inflammatory and other anti-osteoporotic properties [120], showing immunomodulatory activity, since the pectic polysaccharides of this species stimulate [121], platelet anti-aggregation [122] and spasmolytic activity [123].

The functional components of *A. schoenoprasum* are valued for their healing, food, and antimicrobial properties; this is perhaps related to their antioxidant activity [124]. In *A. ampeloprasum*, antioxidant activity is an effect demonstrated by several authors [125,126,127,128]. These studies are carried out through alcoholic extractions of this species, demonstrating their effectiveness both as hypolipidemic and antioxidant, however, as mentioned, the compounds involved in these mechanisms are unknown.

The high content of allicin in leaf extracts of *A. schoenoprasum* may explain the anti-inflammatory effect, in addition to compounds also present in this species such as β-sitosterol and campesterol [129].

Extracts from leaves of *A. humile* and *A. hirtifolium* are rich in sulfur compounds. *A. humile* has cardio-protective effect related to metabolites such as ajoene, allicin, and alliin [130,131], decreasing the risk factors of cardiovascular accidents [132].

Garlic can accumulate up to five times more selenium (110–150 mg·kg^−1^ vs. 28 mg·kg^−1^) and constitutes a more potent anticarcinogen natural agent than onion [25]. Regarding selenium in animal organisms, the MeSeCys ingested with food or administered in supplements is absorbed and distributed more effectively than inorganic Se, and is metabolized to methyl selenol, the chemical species to which anticarcinogenic and antioxidant properties are attributed [133].

In all cases, the potent anti-cancer effect is a result of the presence of Se [134], finding that the selenium-analogs of the sulfur compounds of *Allium*, such as diallyl selenide vs. diallyl sulfide and benzyl selenocyanate vs. benzyl thiocyanate, are often more effective as anticarcinogenic agents [135].

## 4. Use of Selenium and Sulfur in *Allium* Agricultural Production

As mentioned earlier, selenium consumption is of utmost importance for human health. It has been proven that the consumption of this element by humans is mainly given by food since they contribute up to 80% of Se intake [148]. In turn, the natural selenium content in food depends on the geological variations of the surface of the Earth. In most atmospheric conditions, exposure to this element is negligible, as air Se concentrations are <10 ng·m^−3^. In most cases, the content of Se in water is <10 µg·L^−1^, a value considered extremely low, while in seawater the average concentration is 0.09 µg·L^−1^. In the same way, the amount of Se in most soils is very low, ranging from 0.01 to 2 mg·kg^−1^, while the overall mean is 0.4 mg·kg^−1^ [149]. In some regions of Europe, Africa, China, and Thailand, for example, most soils have low concentrations of Se, which results in low concentrations in food crops [150,151,152].

Sulfur, on the other hand, is essential for plant and human metabolism, for example, forming part of amino acids, proteins, and coenzymes [153]. However, in the last 30 years, the availability of this element has declined due to the use of fertilizers with low S content, such as MAP or DAP [154], the progression of intensive agriculture that decreases the major source of sulfur in soil: soil organic matter, as well as the reduction of S in pesticides [155]. There is a need for additional sulfur applications in crops, particularly in highly sulfur-demanding crops such as *Allium* sp. Sulfur fertilization can be carried out through different routes: elemental sulfur, sprinkled in leaves or soil applied, and calcium sulfate incorporated in soil are inexpensive sources of S, which provides a long-term residual effect, especially in clay soils [156].

The main proposal of this paper is to use *Allium* species as specialist plants for biofortification with Se and S. The metabolism of these plants is adapted to this purpose considering the natural ability to accumulate both elements in the form of different phytochemicals, which promotes the functional value of *Allium*. In *Allium* crops grown in soils low in organic matter (< 1%) it is advisable to provide elemental sulfur applied to the soil (30–60 kg·ha^−1^) in addition to the sulfate that fertilizers contain. On the other hand, it is suggested to spray foliar sulfur (2 to 5 kg·ha^−1^ of potassium sulfate or 10 to 20 kg·ha^−1^ of micronized elemental sulfur) on two or three occasions during the growing season, thus avoiding leaching and volatilization of S on the soil as well as bringing the element directly to the site where it will be assimilated and accumulated in organic forms [157]. In the case of selenium, it has also been found that leaf aspersion is the most effective way of biofortifying crops, thus being possible to sprinkle nutrient solutions with sulfate and with selenate or selenite (5–15 g·ha^−1^).

In order to increase the final concentration of biotransformed Se in *Allium* crops, it is recommended to apply selenite or SeMet in concentrations up to 10 mg·L^−1^ of selenium in the nutrient solution or 10 to 50 mg·L^−1^ per leaf (sprinkling a volume of 50 mL·m^−2^). When applied by the irrigation system, it is preferably done once or twice during the growing season and at most once every 15 days [158]. Foliar application is done once when plants have 7–8 leaves [159]. In some species, foliar application of selenite has been found to be the most effective way to obtain biotransformed selenium in plant tissues [160] and would therefore be recommended for *Allium*. The application of Se as a pre-treatment in seeds (using 10–50 g of selenium applied to the seeds needed for one hectare) is another effective way to increase the concentration of Se in seedlings and adult plants. Although information on the use of selenium applied to *Allium* seeds or bulbs is not available, it has been a simple and effective way to apply it [161] and has favorable effects such as increasing the rate of germination under unfavorable conditions [162]. The feasibility of using selenium-enriched substrates has been demonstrated in the seedling stage, which avoids the disadvantages of the dosage in the nutrient solution [163].

Both Se and S are important determinants of the nutraceutical value of *Allium* [164]. However, in many cases, selenium competes with sulfur for root absorption sites because the sulfur form that is absorbed by the roots, SO_4_^2−^, is taken by the same selenate-absorbing transporters (SeO_4_^2−^), which is the most common form of selenium in aerobic soils (low in organic matter, and pH in alkaline side). In contrast, selenite will be the predominant form of selenium in aerobic soils with pH in acidic to neutral side. Selenite does not compete with sulfate, since its absorption is partially mediated by phosphate and silicon transporters [165,166,167] and phosphate-selenite antagonism is found to be much smaller than sulfate-selenate antagonism [168].

### Use of Se and S in Allium Production Systems

Regarding the agronomic management, the capacity of these plants to assimilate sulfur and selenium can be promoted using an adequate level of organic matter in soil [154,169], and a proper balance of S:P:Se; that is, using selenate when sulfate is not in high concentration or using selenite if a large amount of sulfate is found, but providing an adequate amount of phosphates. Thus, the competition for the sites of absorption and subsequent metabolism would not be significant, and both sulfate and selenate or selenite will be rapidly metabolized and incorporated into various compounds with nutraceutical value for humans and increasing the plant ability to tolerate environmental stress [170], through the capacity of sulfur and selenium compounds to promote antioxidant activity and a reduced cellular-redox environment, as well as to coordinate heavy metal and metalloid ions, diminishing oxidative stress and damage to DNA [171,172].

Several studies highlight the benefits of Se in the production of *Allium* (Table 2). The application of Se relates directly to the antioxidant capacity and functional value of *Allium* [173,174,175]. In addition to increasing nutritional quality, increased antioxidant capacity would result in a potential increase in the plant’s ability to tolerate stress. The application of Se in plants increases biomass accumulation [176] or yield [159]. However, high concentrations (10 and 100 mg·L^−1^ of selenate or selenite in the nutrient solution) inhibit growth in garlic [177]. Growth inhibition in hydroponics was reported in onion with selenate at 2 mg·L^−1^ [50,177], with 5–100 mg·L^−1^ of selenate or selenite in the nutrient solution [173], or with 50 mg of selenate per kg of soil [178].

Positive effects of Se have been reported, such as Hg antagonism [179], and even the ability to decrease Hg toxicity when the plants are simultaneously exposed to both elements [180]. Selenium also presents antagonism with other nutrients such as Ca and K [175], and particularly with S [18,31,141,175]. In garlic it has been shown that the application of S can inhibit the uptake of Se [141], whereas the Se at high concentration (50–100 mg·L^−1^ by leaf spraying) decreases the absorption of S [175]. However, there is not always an antagonism between these elements. In different cultivars of onion it was observed that the application of Na_2_SeO_4_ in nutrient solution (up to 2 mg·L^−1^) generated an increase in S concentration [181] indicating the possibility of applying both elements, but with the adequate concentration of each one of them. When comparing selenite and selenate application in *Allium*, consistently higher selenite toxicity has been observed [177], and increased selenium accumulation when applied as selenate [65,182]. More selenium was accumulated in *A. schoenoprasum* when selenate was applied, in comparison to selenite and SeMet, whereas in *A. fistulosum* greater accumulation of the element was observed when using SeMet compared to selenite. However, considering the objective of applying S and Se together, the best way to use selenium in *Allium* plants would be selenite > SeMet > selenate, specifically using a low concentration of Se (≤2 mg·L^−1^ or ≤5 mg·kg^−1^ of soil) (Table 2). Selenium at low level results in positive effects on antioxidant capacity and growth, without negatively affecting the assimilation of sulfur.

Sulfur exerts well-documented benefits in *Allium* (Table 3), mainly on biomass and bulb size [142,183] as well as other related characteristics as leaf number, plant height, and yield [184,185,186]. Another example is the positive relationship between sulfur availability and pungency [31,142,183,184,187,188,189,190,191], whereas low levels of S decrease pungency [192,193]. However, the effect of S on pungency may be variety dependent, as observed in onion [29].

Regarding the interaction of S with other elements, it is well known its antagonistic effect with Se as previously discussed. However, the effect is not exclusive to Se. In onion, S antagonism has been demonstrated with B, Fe, Mn and Zn [193], whereas in garlic with Cl and Na [194]. However, a synergistic effect on other elements has also been reported. In garlic the foliar application of S increased the content of N, P and K [194], whereas in *A. fistulosum* application in nutrient solution increased the N content [184].

## 5. Conclusions

*Allium* crops are suitable models for the combined biofortification with sulfur and selenium because these plants have a high capacity for absorption, transport, and biotransformation to obtain phytochemicals that determine the nutraceutical value of the crops. The amounts to be applied and the chemical forms for the application of sulfur and selenium were explained in the text, highlighting the impact on quality and productivity.

## Figures and Tables

**Table 1:** The impact of the biofortification with Se and S on some phytochemicals of *Allium* plants.

Biofortification	Phytochemical	References
S in *A. roseum*	Diallyl disulfide	[136]
S in *A. roseum*	Diallyl thiosulfinate (Allicin)	[136]
S in *A. roseum*	Methyl allyl disulfide	[136]
Se in *A. tricoccum*	Se-methylselenocysteine	[137]
Se in garlic and *A. ascalunicum*	γ-glutamyl-Se-methlyselenocysteine (γ-GluMeSeCys)	[138]
Se in garlic and chives	γ-glutamyl-Se-methlyselenocysteine (γ-GluMeSeCys)	[139,140]
Se in garlic and chives	Se-methylselenocysteine	[140,141]
S in onion	γ-glutamyl-1-propenyl cysteine sulfoxide (γGPECSO)	[142]
S in onion	Propyl cysteine sulfoxide (Propiin)	[143]
S in onion	*S*-methyl alkyl cysteine sulfoxides (Mettin)	[143]
S in onion	*S*-methyl-l-cysteine sulfoxide	[144]
S in onion	Trans-*S*-1-propenyl-l-cysteine sulfoxide (1-PRENCSO)	[142]
Se in onion	*S*-methyl-l-cysteine sulfoxide	[50]
S in onion and garlic	Propenyl cysteine sulfoxide (Isoalliin)	[145,146]
S in onion and garlic	*S*-allyl cysteine sulfoxide (Aliin)	[145,147]

**Table 2:** Effects of selenium application on *Allium* species selenium content, growth, yield, and quality.

Species	Chemical Form	Application Form	Quantity Supplied	Results	Reference
Chives	Na_2_SeO_3_	Nutrient solution	30 mg·L^−1^	Accumulation of Se in root > leaf. Antagonism with Hg.	[179]
Chives	Na_2_SeO_3_	Soil	1, 2, 3, 5 and 15 mg·kg^−1^ Se	Increases Se content.	[140]
Chives	Se(IV), SeMet	Nutrient solution	10 and 100 mg·L^−1^	Higher stress tolerance and Se accumulation with SeMet.	[195]
Elephant garlic	Na_2_SeO_3_, Na_2_SeO_4_	Soil	0.2, 1.3, 2.6 y 3.8 mg·kg^−1^ Se	Accumulation of selenate > selenite.	[65]
Garlic	K_2_SeO_3_ y K_2_SeO_4_	Hydroponics	50 µmol·L^−1^	Increases Se content. Antagonism with S.	[141]
Garlic	Na_2_SeO_3_	Hydroponics	3 and 6 µmol·L^−1^	Low dose increases biomass and delays senescence.	[176]
Garlic	Na_2_SeO_4_	Leaf spray	10, 50 and 100 mg·L^−1^	Antagonism with S, K and Ca. Increases antioxidant capacity.	[175]
Garlic	Na_2_SeO_3_, Na_2_SeO_4_	Nutrient solution	0.01, 0.1, 1, 10, 100 mg·L^−1^	Inhibition of growth in high doses. Decreases Hg toxicity.	[180]
Onion	Na_2_SeO_3_	Soil and foliar spray	10, 20 and 40 kg·ha^−1^ on soil. 0.5, 1, 1.5, 2, 2.5 and 3 mg·L^−1^ leaf spray	Antagonism with S.	[31]
Onion	Na_2_SeO_4_	Foliar spray	10, 50 and 100 mg·L^−1^	Antagonism with S. 50 μg·mL^−1^ increases Se content.	[159]
Onion	Na_2_SeO_4_	Hydroponics	1, 2, 4 and 8 mg·L^−1^ SeO_4_	Increases Se content. Antagonism with S.	[18]
Onion	Na_2_SeO_4_	Hydroponics	2.0 mg L^−1^	Increases Se content.	[50]
Onion	Na_2_SeO_4_	Hydroponics	0.5, 1.0, 1.5 and 2.0 mg·L^−1^	High concentrations decrease growth. Low concentration increases S content.	[181]
Onion	Na_2_SeO_3_, Na_2_SeO_4_	Nutrient solution	5 mg·L^−1^	Affects growth. Bulb accumulation > leaf > root.	[173]
Onion	Na_2_SeO_3_ and Na_2_SeO_4_	Soil	2.5, 5.0 and 7.5 mg·kg^−1^	Increase the content of Se. Decreases growth. Selenite is more beneficial than selenate.	[196]
Onion	Se(VI)	Soil	25 and 50 mg·kg^−1^	Accumulation of Se, high dose decreases bulb size.	[178]
Scallion	Selenite, selenate, SeMet	Nutrient solution	10 mg·L^−1^	Higher concentration of Se with selenate. 30% of the Se is inorganic.	[182]

**Table 3:** Effects of sulfur application on *Allium* quality and functional value depending on the chemical form, application form and quantity supplied.

Species	Chemical Form	Application Form	Quantity Supplied	Results	Reference
Chives	K_2_SO_4_	Soil	60 mg·kg^−1^ soil	Increased S content.	[197]
Chives	K_2_SO_4_, MgSO_4_ and H_2_SO_4_	Nutrient solution	0.01 and 4.0 mmol·L^−1^ SO_4_	Increased biomass, N, S, and pungency.	[184]
Chives	MgSO_4_ and K_2_SO_4_	Soil	0.1, 1.75 and 4.0 mM SO_4_	Increased content of pyruvate, S, and dry weight.	[191]
Onion	CaSO_4_	Hydroponics	0.8, 4.8, 10.8 and 14.8 mol·m^−3^	Increased concentration of S. Antagonism with Se.	[18]
Onion	CaSO_4_	Soil	22.4 kg·ha^−1^ S	No effects.	[198]
Onion	CaSO_4_	Soil	200 kg·ha^−1^ S	Increased S, pungency, and pyruvic acid concentration.	[189]
Onion	CaSO_4_	Soil	20, 40 and 60 kg·ha^−1^ S	Up to 40 kg ha^−1^ increased growth and yield.	[199]
Onion	Elemental sulfur (Sulfurgran^®^)	Soil	15, 30, 45, 60 and 90 kg·ha^−1^	Improves growth and yield.	[200]
Onion	H_2_SO_4_	Nutrient solution	0.1 and 4.0 meq·L^−1^	Increased pungency and concentration of S.	[187]
Onion	H_2_SO_4_	Nutrient solution	0.1 and 4.0 meq·L^−1^	Increased pungency and concentration of S.	[188]
Onion	K_2_Mg_2_(SO_4_)_3_	Soil	30 and 50 kg·ha^−1^ S	Increased S and pyruvate content.	[143]
Onion	K_2_O_3_S_2_	Soil	80, 126, 172, 218 and 264 kg·ha^−1^ S	No effect on growth and yield.	[201]
Onion	Liquid sulfur (17%)	Soil surface	13 and 26 kg·ha^−1^	No effect.	[202]
Onion	MgSO_4_	Nutrient solution	0.1 and 4.0 meq·L^−1^	Increased pyruvic acid and S concentration.	[203]
Onion	MgSO_4_	Nutrient solution	0.1, 0.48, 0.85, 1.6, and 3.1 meq·L^−1^	Increased bulb fresh weight up to 1.6 meq L^−1^.	[204]
Onion	MgSO_4_	Nutrient solution	5, 25, 50, 75 and 150 mg·L^−1^	Increased total S and sulfates. Differences in varieties in pyruvic acid.	[29]
Onion	MgSO_4_	Nutrient solution	5, 45 and 125 mg·L^−1^	S application increased bulb weight.	[142]
Onion	MgSO_4_	Nutrient solution	1.7, 15 and 41.7 mg·L^−1^	Positive effect in pungency. Antagonism with B, Fe, Mn, and Zn.	[193]
Onion	MgSO_4_ and CaSO_4_	Nutrient solution	2 and 123 mg·L^−1^	S in leaves, pungency, and yield decreases with low S level.	[192]
Onion	Na_2_SO_4_	Soil	15, 30 and 45 kg·ha^−1^ S	Increased pyruvic acid. Antagonism with Se.	[31]

## References

[1] Benton Jones J. (2012). Plant Nutrition and Soil Fertility Manual.

[2] Haraguchi H. (2004). Metallomics as integrated biometal science. J. Anal. At. Spectrom..

[3] Koca A., Koca I., Tekguler B. (2016). Two antoxidant elements of *Allium* vegetables: Germanium and Selenium. Acta Hortic..

[4] Reilly K., Valverde J., Finn L., Gaffney M., Rai D.K., Brunton N. (2014). A note on the effectiveness of Selenium supplementation of Irish-grown *Allium* crops. Irish J. Agric. Food Res..

[5] Pilon-Smits E.A.H., Bañuelos G.S., Parker D.R., Chang A.C., Brawer Silva D. (2014). Uptake, Metabolism, and Volatilization of Selenium by Terrestrial Plants. Salinity and Drainage in San Joaquin Valley, California: Science, Technology, and Policy.

[6] Slekovec M., Goessler W. (2005). Accumulation of Selenium in natural plants and Selenium supplemented vegetable and Selenium speciation by HPLC-ICPMS. Chem. Speciat. Bioavailab..

[7] Cooke J., DeGabriel J.L. (2016). Editorial: Plant silicon interactions between organisms and the implications for ecosystems. Front. Plant Sci..

[8] Medrano-Macías J., Leija-Martínez P., González-Morales S., Juárez-Maldonado A., Benavides-Mendoza A. (2016). Use of iodine to biofortify and promote growth and stress tolerance in crops. Front. Plant Sci..

[9] Jones G.D., Droz B., Greve P., Gottschalk P., Poffet D., Mcgrath S.P., Seneviratne S.I., Smith P., Winkel L.H. (2017). Selenium deficiency risk predicted to increase under future climate change. Proc. Natl. Acad. Sci. USA.

[10] O’Donnell G., Gibbons S. (2007). Antibacterial activity of two canthin-6-one alkaloids from *Allium neapolitanum*. Phyther. Res..

[11] Griffiths G., Trueman L., Crowther T., Thomas B., Smith B. (2002). Onions—A global benefit to health. Phyther. Res..

[12] Lundegårdh B., Botek P., Schulzov V., Hajšlov J., Strömberg A., Andersson H.C. (2008). Impact of different green manures on the content of *S*-alk(en)yl-l-cysteine sulfoxides and l-ascorbic acid in leek (*Allium porrum*). J. Agric. Food Chem..

[13] Broadley M.R., White P.J., Bryson R.J., Meacham M.C., Bowen H.C., Johnson S.E., Hawkesford M.J., McGrath S.P., Zhao F.-J., Breward N. (2006). Biofortification of UK food crops with Selenium. Proc. Nutr. Soc..

[14] Meija J., Montes-Bayón M., Le Duc D.L., Terry N., Caruso J.A. (2002). Simultaneous monitoring of volatile selenium and sulfur species from Se accumulating plants (Wild type and genetically modified) by GC/MS and GC/ICPMS using solid-phase microextraction for sample introduction. Anal. Chem..

[15] De Souza M.P., Pilon-Smits E.A.H., Lytle C.M., Hwang S., Tai J., Honma T.S.U., Yeh L., Terry N. (1998). Rate-limiting steps in Selenium assimilation and volatilization by Indian mustard. Plant Physiol..

[16] Terry N., Carlson C., Raab T.K., Zayed A.M. (1992). Rates of Selenium volatilization among crop species. J. Environ. Qual..

[17] Zayed A.M., Terry N. (1992). Selenium volatilization in broccoli as influenced by sulfate supply. J. Plant Physiol..

[18] Barak P., Goldman I.L. (1997). Antagonistic relationship between selenate and sulfate uptake in onion (*Allium cepa*): Implications for the production of organosulfur and organoselenium compounds in plants. J. Agric. Food Chem..

[19] Pilon-Smits E.A., Quinn C.F., Tapken W., Malagoli M., Schiavon M. (2009). Physiological functions of beneficial elements. Curr. Opin. Plant Biol..

[20] Stroud J.L., Broadley M.R., Foot I., Fairweather-Tait S.J., Hart D.J., Hurst R., Knott P., Mowat H., Norman K., Scott P. (2010). Soil factors affecting Selenium concentration in wheat grain and the fate and speciation of Se fertilisers applied to soil. Plant Soil.

[21] Hu Q., Chen L., Xu J., Zhang Y., Pan G. (2002). Determination of Selenium concentration in rice and the effect of foliar application of Se-enriched fertiliser or sodium selenite on the Selenium content of rice. J. Sci. Food Agric..

[22] Kotrebai M., Birringer M., Tyson J.F., Block E., Uden P.C. (2000). Selenium speciation in enriched and natural samples by HPLC-ICP-MS and HPLC-ESI-MS with perfluorinated carboxylic acid ion-pairing agents. Analyst.

[23] Izgi B., Gucer S., Jaćimović R. (2006). Determination of Selenium in garlic (*Allium sativum*) and onion (*Allium cepa*) by electro thermal atomic absorption spectrometry. Food Chem..

[24] Giacosa A., Faliva M., Perna S., Minoia C., Ronchi A., Rondanelli M. (2014). Selenium fortification of an italian rice cultivar via foliar fertilization with sodium selenate and its effects on human serum Selenium levels and on erythrocyte glutathione peroxidase activity. Nutrients.

[25] Ip C., Lisk D.J. (1994). Enrichment of Selenium in allium vegetables for cancer prevention. Carcinogenesis.

[26] Whanger P.D., Ip C., Polan C.E., Uden P.C., Welbaum G. (2000). Tumorigenesis, metabolism, speciation, bioavailability, and tissue deposition of Selenium in Selenium-enriched ramps (*Allium tricoccum*). J. Agric. Food Chem..

[27] Haneklaus S., Bloem E., Schnug E., De Kok L.J., Stulen I., Barker A.V., Pilbeam D.J. (2007). Sulfur. Handbook of Plant Nutrition.

[28] Minard H.R.G. (1978). Effect of clove size, spacing, fertilisers, and lime on yield and nutrient content of garlic (*Allium sativum*). N. Z. J. Exp. Agric..

[29] Randle W.M., Kopsell D.E., Kopsell D.A., Snyder R.L. (1999). Total sulfur and sulfate accumulation in onion is affected by sulfur fertility. J. Plant Nutr..

[30] Kabata-Pendias A. (2011). Trace Elements in Soils and Plants.

[31] Qureshi A.A., Lawande K.E., Patil V.B., Mani S. (2012). Relationship between Selenium and Sulfur assimilation and resultant interaction on quality parameters in onion. Commun. Soil Sci. Plant Anal..

[32] El-Ramady H.R., Domokos-Szabolcsy É., Shalaby T.A., Prokisch J., Fári M., Lichtfouse E., Schwarzbauer J., Robert D. (2015). Selenium in agriculture: Water, air, soil, plants, food, animals and nanoselenium. CO_2_ Sequestration, Biofuels and Depollution.

[33] Suzuki I. (1999). Oxidation of inorganic sulfur compounds: Chemical and enzymatic reactions. Can. J. Microbiol..

[34] Droux M. (2004). Sulfur assimilation and the role of Sulfur in plant metabolism: A survey. Photosynth. Res..

[35] Kertesz M.A., Mirleau P. (2004). The role of soil microbes in plant Sulphur nutrition. J. Exp. Bot..

[36] Lucheta A., Lambais M. (2012). Sulfur in agriculture. Rev. Bras. Ciência do Solo.

[37] Griffith C.M., Woodrow J.E., Seiber J.N. (2015). Environmental behavior and analysis of agricultural Sulfur. Pest Manag. Sci..

[38] White P.J., Bowen H.C., Parmaguru P., Fritz M., Spracklen W.P., Spiby R.E., Meacham M.C., Mead A., Harriman M., Trueman L.J. (2004). Interactions between Selenium and sSlphur nutrition in *Arabidopsis thaliana*. J. Exp. Bot..

[39] El-Ramady H., Abdalla N., Alshaal T., El-Henawy A., Faizy S.E.D.A., Shams M.H., Shalaby T., Bayoumi Y., Elhawat N., Shehata S., Lichtfouse E., Schwarzbauer J., Robert D. (2015). Selenium and its role in higher plants. Pollutants in Buildings, Water and Living Organisms.

[40] Terry N., Zayed A.M., de Souza M.P., Tarun A. (2000). Selenium in higher plants. Annu. Rev. Plant Physiol. Plant Mol. Biol..

[41] Arnault I., Auger J. (2006). Seleno-compounds in garlic and onion. J. Chromatogr. A.

[42] El-Ramady H., Abdalla N., Taha H.S., Alshaal T., El-Henawy A., Faizy S.E.D.A., Shams M.S., Youssef S.M., Shalaby T., Bayoumi Y. (2016). Selenium and nano-selenium in plant nutrition. Environ. Chem. Lett..

[43] Buchner P., Jaiwal P.K., Singh R.P., Dhankher O.P. (2008). Plant sulfate transporters. Plant Membrane and Vacuolar Transporters.

[44] Hawkesford M.J. (2003). Transporter gene families in plants: The sulphate transporter gene family—Redundancy or specialization?. Physiol. Plant..

[45] Hawkesford M.J., De Kok L.J. (2006). Managing Sulphur metabolism in plants. Plant Cell Environ..

[46] Maruyama-Nakashita A., Inoue E., Watanabe-Takahashi A., Yamaya T., Takahashi H. (2003). Transcriptome profiling of Sulfur-responsive genes in *Arabidopsis* reveals global effects of Sulfur nutrition on multiple metabolic pathways. Plant Physiol..

[47] Takahashi H., Buchner P., Yoshimoto N., Hawkesford M.J., Shiu S.-H. (2012). Evolutionary relationships and functional diversity of plant sulfate transporters. Front. Plant Sci..

[48] Shibagaki N., Rose A., McDermott J.P., Fujiwara T., Hayashi H., Yoneyama T., Davies J.P. (2002). Selenate-resistant mutants of *Arabidopsis thaliana* identify Sultr1;2, a sulfate transporter required for efficient transport of sulfate into roots. Plant J..

[49] Van Hoewyk D., Takahashi H., Inoue E., Hess A., Tamaoki M., Pilon-Smits E.A.H. (2008). Transcriptome analyses give insights into Selenium-stress responses and Selenium tolerance mechanisms in *Arabidopsis*. Physiol. Plant..

[50] Kopsell D.A., Randle W.M. (1999). Selenium affects the *S*-alk(en)yl cysteine sulfoxides among short-day onion cultivars. J. Am. Soc. Hortic. Sci..

[51] Cherest H., Davidian J.-C., Thomas D., Benes V., Ansorge W., Surdin-Kerjan Y. (1997). Molecular characterization of two high affinity sulfate transporters in *Saccharomyces cerevisiae*. Genetics.

[52] Takahashi H., Watanabe-Takahashi A., Smith F.W., Blake-Kalff M., Hawkesford M.J., Saito K. (2000). The roles of three functional sulphate transporters involved in uptake and translocation of sulphate in *Arabidopsis thaliana*. Plant J..

[53] Yoshimoto N., Takahashi H., Smith F.W., Yamaya T., Saito K. (2002). Two distinct high-affinity sulfate transporters with different inducibilities mediate uptake of sulfate in *Arabidopsis* roots. Plant J..

[54] Kataoka T., Watanabe-Takahashi A., Hayashi N., Ohnishi M., Mimura T., Buchner P., Hawkesford M.J., Yamaya T., Takahashi H. (2004). Vacuolar sulfate transporters are essential determinants controlling internal distribution of sulfate in *Arabidopsis*. Plant Cell.

[55] Yoshimoto N., Inoue E., Saito K., Yamaya T., Takahashi H. (2003). Phloem-localizing sulfate transporter, Sultr1;3, mediates re-distribution of Sulfur from source to sink organs in *Arabidopsis*. Plant Physiol..

[56] Kopriva S., Calderwood A., Weckopp S.C., Koprivova A. (2015). Plant Sulfur and Big Data. Plant Sci..

[57] Lopez J., Bell C.I., Tremblay N., Dorais M., Gosselin A. (2002). Uptake and translocation of sulphate in tomato seedlings in relation to sulphate supply. J. Plant Nutr..

[58] Briggs W.H., Goldman I.L. (2002). Variation in economically and ecologically important traits in onion plant organs during reproductive development. Plant. Cell Environ..

[59] Durenkamp M., De Kok L.J. (2004). Impact of pedospheric and atmospheric Sulphur nutrition on Sulphur metabolism of *Allium cepa* L., a species with a potential sink capacity for secondary Sulphur compounds. J. Exp. Bot..

[60] Pandey C., Gupta M. (2015). Selenium and auxin mitigates Arsenic stress in rice (*Oryza sativa* L.) by combining the role of stress indicators, modulators and genotoxicity assay. J. Hazard. Mater..

[61] Pazurkiewicz-Kocot K., Kita A., Pietruszka M. (2008). Effect of Selenium on Magnesium, Iron, Manganese, Copper, and Zinc accumulation in corn treated by Indole-3-acetic acid. Commun. Soil Sci. Plant Anal..

[62] Cao M.J., Wang Z., Wirtz M., Hell R., Oliver D.J., Xiang C. (2013). Bin SULTR3;1 is a chloroplast-localized sulfate transporter in *Arabidopsis thaliana*. Plant J..

[63] Cao M.J., Wang Z., Zhao Q., Mao J.L., Speiser A., Wirtz M., Hell R., Zhu J.K., Xiang C. (2014). Bin Sulfate availability affects ABA levels and germination response to ABA and salt stress in *Arabidopsis thaliana*. Plant J..

[64] Bulska E., Wierzbicka I.A., Wysocka M.H., Proost K., Janssens K., Falkenberg G. (2006). In vivo investigation of the distribution and the local speciation of Selenium in *Allium cepa* L. by means of microscopic X-ray Absorption Near-Edge Structure Spectroscopy and Confocal Microscopic X-ray Fluorescence Analysis. Anal. Chem..

[65] Lavu R.V.S., Du Laing G., Van De Wiele T., Pratti V.L., Willekens K., Vandecasteele B., Tack F. (2012). Fertilizing soil with Selenium fertilizers: Impact on concentration, speciation, and bioaccessibility of Selenium in leek (*Allium ampeloprasum*). J. Agric. Food Chem..

[66] Sun X.D., Yu X.H., Zhou S.M., Liu S.Q. (2016). De novo assembly and characterization of the welsh onion (*Allium fistulosum* L.) transcriptome using Illumina technology. Mol. Genet. Genom..

[67] Chao D.-Y., Baraniecka P., Danku J., Koprivova A., Lahner B., Luo H., Yakubova E., Dilkes B., Kopriva S., Salt D.E. (2014). Variation in sulfur and selenium accumulation is controlled by naturally occurring isoforms of the key Sulfur assimilation enzyme ADENOSINE 5’-PHOSPHOSULFATE REDUCTASE2 across the *Arabidopsis* species range. Plant Physiol..

[68] McCallum J.A., Pither-Joyce M., Shaw M. (2002). Sulfur deprivation and genotype affect gene expression and metabolism of onion roots. J. Am. Soc. Hortic. Sci..

[69] McManus M.T., Joshi S., Searle B., Pither-Joyce M., Shaw M., Leung S., Albert N., Shigyo M., Jakse J., Havey M.J. (2012). Genotypic variation in Sulfur assimilation and metabolism of onion (*Allium cepa* L.) III. Characterization of sulfite reductase. Phytochemistry.

[70] Feldman-Salit A., Wirtz M., Hell R., Wade R.C. (2009). A mechanistic model of the Cysteine Synthase Complex. J. Mol. Biol..

[71] Novoselov S.V., Rao M., Onoshko N.V., Zhi H., Kryukov G.V., Xiang Y., Weeks D.P., Hatfield D.L., Gladyshev V.N. (2002). Selenoproteins and selenocysteine insertion system in the model plant cell system, *Chlamydomonas reinhardtii*. EMBO J..

[72] Jones M.G., Hughes J., Tregova A., Milne J., Tomsett A.B., Collin H.A. (2004). Biosynthesis of the flavour precursors of onion and garlic. J. Exp. Bot..

[73] Hughes J., Tregova A., Tomsett A.B., Jones M.G., Cosstick R., Collin H.A. (2005). Synthesis of the flavour precursor, alliin, in garlic tissue cultures. Phytochemistry.

[74] Edmands W.M.B., Gooderham N.J., Holmes E., Mitchell S.C. (2013). *S*-Methyl-l-cysteine sulphoxide: The Cinderella phytochemical?. Toxicol. Res..

[75] Otte M.L., Wilson G., Morris J.T., Moran B.M. (2004). Dimethylsulphoniopropionate (DMSP) and related compounds in higher plants. J. Exp. Bot..

[76] Charlson R.J., Lovelock J.E., Andreae M.O., Warren S.G. (1987). Oceanic phytoplankton, atmospheric Sulphur, cloud albedo and climate. Nature.

[77] Van Hoewyk D., Garifullina G.F., Ackley A.R., Abdel-Ghany S.E., Marcus M.A., Fakra S., Ishiyama K., Inoue E., Pilon M., Takahashi H. (2005). Overexpression of AtCpNifS enhances Selenium tolerance and accumulation in *Arabidopsis*. Plant Physiol..

[78] Bloem E., Riemenschneider A., Volker J., Papenbrock J., Schmidt A., Salac I., Haneklaus S., Schnug E. (2004). Sulphur supply and infection with *Pyrenopeziza brassicae* influence l-cysteine desulphydrase activity in *Brassica napus* L. J. Exp. Bot..

[79] Rausch T., Wachter A. (2005). Sulfur metabolism: A versatile platform for launching defence operations. Trends Plant Sci..

[80] Dini I., Tenore G.C., Dini A. (2008). *S*-Alkenyl Cysteine Sulfoxide and Its antioxidant properties from *Allium cepa* var. tropeana (Red Onion) seeds. J. Nat. Prod..

[81] Rose P., Whiteman M., Moore K., Zhun Y. (2005). Bioactive *S*-alk(en)yl cysteine sulfoxide metabolites in the genus *Allium*: The chemistry of potential therapeutic agents. Nat. Prod. Rep..

[82] Dugravot S., Brunissen L., Létocart E., Tjallingii W.F., Vincent C., Giordanengo P., Cherqui A. (2007). Local and systemic responses induced by aphids in *Solanum tuberosum* plants. Entomol. Exp. Appl..

[83] Varin L., Marsolais F., Richard M., Rouleau M. (1997). Sulfation and sulfotransferases 6: Biochemistry and molecular biology of plant sulfotransferases. FASEB J..

[84] Riemenschneider A., Riedel K., Hoefgen R., Papenbrock J., Hesse H. (2005). Impact of reduced *O*-Acetylserine(thiol)lyase Isoform contents on potato plant metabolism. Plant Physiol..

[85] Randle W.M., Lancaster J.E., Rabinowitch H.D., Currah L. (2002). Sulphur compounds in Alliums in relation to flavour quality. Allium Crop Science: Recent Advances.

[86] Yoshimoto N., Yabe A., Sugino Y., Murakami S., Sai-Ngam N., Sumi S.-I., Tsuneyoshi T., Saito K. (2015). Garlic γ-glutamyl transpeptidases that catalyze deglutamylation of biosynthetic intermediate of alliin. Front. Plant Sci..

[87] Auger J., Yang W., Arnault I., Pannier F., Potin-Gautier M. (2004). High-performance liquid chromatographic-inductively coupled plasma mass spectrometric evidence for Se-“alliins” in garlic and onion grown in Se-rich soil. J. Chromatogr. A.

[88] Rasmussen R.A. (1974). Emission of biogenic hydrogen sulfide. Tellus.

[89] Vairavamurthy A., Andreae M.O., Iverson R.L. (1985). Biosynthesis of dimethlysulfide and dimethylpropiothetin by *Hymenomonas carterae* in relation to Sulfur source and salinity variations. Limnol. Oceanogr..

[90] Calderwood A., Kopriva S. (2014). Hydrogen sulfide in plants: From dissipation of excess Sulfur to signaling molecule. Nitric Oxide.

[91] Kelly D. (1994). The evolutionary ecology of mast seeding. Trends Ecol. Evol..

[92] Wilson L.G., Bressan R.A., Filner P. (1978). Light-dependent mmission of Hydrogen Sulfide from plants. Plant Physiol..

[93] Sekiya J., Wilson L.G., Filner P. (1982). Resistance to Injury by Sulfur Dioxide: Correlation with Its Reduction to, and Emission of, Hydrogen Sulfide in Cucurbitaceae. Plant Physiol..

[94] Bloem E., Haneklaus S., Salac I., Wickenhäuser P., Schnug E. (2007). Facts and fiction about Sulfur metabolism in relation to plant-pathogen interactions. Plant Biol..

[95] Papenbrock J., Riemenschneider A., Kamp A., Schulz-Vogt H.N., Schmidt A. (2007). Characterization of cysteine-degrading and H2S-releasing enzymes of higher plants-from the field to the test tube and back. Plant Biol..

[96] Andreae M.O. (1990). Ocean-atmosphere interactions in the global biogeochemical Sulfur cycle. Mar. Chem..

[97] Andreae M.O., Crutzen P.J. (1997). Atmospheric aerosols: Biogeochemical sources and role in atmospheric chemistry. Science.

[98] Faloona I. (2009). Sulfur processing in the marine atmospheric boundary layer: A review and critical assessment of modeling uncertainties. Atmos. Environ..

[99] Beilstein M.A., Whanger P.D., Yang G.Q. (1991). Chemical forms of Selenium in corn and rice grown in a high Selenium area of China. Biomed. Environ. Sci. BES.

[100] Virupaksha T.K., Shrift A. (1965). Biochemical differences between Selenium accumulator and non-accumulator astragalus species. Biochim. Biophys. Acta Gen. Subj..

[101] Wang Y., Böck A., Neuhierl B. (1999). Acquisition of selenium tolerance by a Selenium non-accumulating *Astragalus* species via selection. Biofactors.

[102] Zhu Y.G., Pilon-Smits E.A.H., Zhao F.J., Williams P.N., Meharg A.A. (2009). Selenium in higher plants: Understanding mechanisms for biofortification and phytoremediation. Trends Plant Sci..

[103] Block E. (1992). The organosulfur chemistry of the genus *Allium*—Implications for the organic chemistry of Sulfur. Angew. Chem. Int. Ed. Engl..

[104] Block E., Gulati H., Putman D., Sha D., You N., Zhao S.-H. (1997). *Allium* chemistry: Synthesis of 1-[alk(en)ylsulfinyl]propyl alk(en)yl disulfides (cepaenes), antithrombotic flavorants from homogenates of onion (*Allium cepa*). J. Agric. Food Chem..

[105] Wagner H., Dorsch W., Bayer T., Breu W., Willer F. (1990). Antiasthmatic effects of onions: Inhibition of 5-lipoxygenase and cyclooxygenase in vitro by thiosulfinates and “Cepaenes”. Prostaglandins Leukot. Essent. Fat. Acids.

[106] Rizwani G.H., Shareef H. (2011). Genus *Allium*: The potential nutritive and therapeutic source. J. Pharm. Nutr. Sci..

[107] Yun H.-M., Ban J.O., Park K.-R., Lee C.K., Jeong H.-S., Han S.B., Hong J.T. (2014). Potential therapeutic effects of functionally active compounds isolated from garlic. Pharmacol. Ther..

[108] Miron T., Rabinkov A., Mirelman D., Wilchek M., Weiner L. (2000). The mode of action of allicin: Its ready permeability through phospholipid membranes may contribute to its biological activity. Biochim. Biophys. Acta (BBA) Biomembr..

[109] Slusarenko A.J., Patel A., Portz D. (2008). Control of plant diseases by natural products: Allicin from garlic as a case study. Eur. J. Plant Pathol..

[110] Mahboubi M., Kazempour N. (2014). Chemical composition, antioxidant and antimicrobial activity of *Allium hirtifolium* essential oil. J. Microbiol. Biotechnol. Food Sci..

[111] Hosono T., Hosono-Fukao T., Inada K., Tanaka R., Yamada H., Iitsuka Y., Seki T., Hasegawa I., Ariga T. (2008). Alkenyl group is responsible for the disruption of microtubule network formation in human colon cancer cell line HT-29 cells. Carcinogenesis.

[112] Xiao D., Choi S., Johnson D.E., Vogel V.G., Johnson C.S., Trump D.L., Lee Y.J., Singh S.V. (2004). Diallyl trisulfide-induced apoptosis in human prostate cancer cells involves c-Jun *N*-terminal kinase and extracellular-signal regulated kinase-mediated phosphorylation of Bcl-2. Oncogene.

[113] Xiao D., Lew K.L., Kim Y.-A., Zeng Y., Hahm E.-R., Dhir R., Singh S.V. (2006). Diallyl Trisulfide suppresses growth of PC-3 human prostate cancer Xenograft In vivo in association with Bax and Bak induction. Clin. Cancer Res..

[114] Sakamoto K., Lawson L.D., Milner J.A. (1997). Allyl sulfides from garlic suppress the in vitro proliferation of human a549 lung tumor cells. Nutr. Cancer.

[115] Xiao D., Pinto J.T., Gundersen G.G., Weinstein I.B. (2005). Effects of a series of organosulfur compounds on mitotic arrest and induction of apoptosis in colon cancer cells. Mol. Cancer Ther..

[116] Sundaram S.G., Milner J.A. (1996). Diallyl disulfide induces apoptosis of human colon tumor cells. Carcinogenesis.

[117] Sparnins V.L., Barany G., Wattenberg L.W. (1988). Effects of organosulfur compounds from garlic and onions on benzo[*a*]pyrene-induced neoplasia and glutathione S-transferase activity in the mouse. Carcinogenesis.

[118] Kazemi S., Asgary S., Moshtaghian J., Rafieian M., Adelnia A., Shamsi F. (2010). Liver-protective effects of hydroalcoholic extract of *Allium hirtifolium* Boiss. in rats with alloxan-induced diabetes mellitus. ARYA Atheroscler. J..

[119] Huang Z., Ren J.W. (2013). Antibacterial activity of elephant garlic and its effect against U2OS human osteosarcoma cells. Iran. J. Basic Med. Sci..

[120] El-Shenawy S.M., Yassin N.A., Badary O.A., EL-Moneem M., AL-Shafeiy H.M. (2013). Study of the effect of *Allium porrum* on osteoporosis induced in rats. Der. Pharm. Lett..

[121] Kratchanova M., Nikolova M., Pavlova E., Yanakieva I., Kussovski V. (2010). Composition and properties of biologically active pectic polysaccharides from leek (*Allium porrum*). J. Sci. Food Agric..

[122] Fattorusso E., Lanzotti V., Taglialatela-Scafati O., Cicala C. (2001). The flavonoids of leek, *Allium porrum*. Phytochemistry.

[123] Sedighi M., Rafieian-Kopaei M., Noori-Ahmadabadi M. (2012). Effect of *Allium ampeloprasum* on ileum function: Involvement of beta-adrenergic receptors and voltage dependent calcium channels. Life Sci. J..

[124] Štajner D., Popović B.M., Ćalić-Dragosavac D., Malenčić Đ., Zdravković-Korać S. (2011). Comparative study on *Allium schoenoprasum* cultivated plant and *Allium* schoenoprasum tissue culture organs antioxidant status. Phyther. Res..

[125] Nguansangiam S., Angsubhakorn S., Bhamarapravati S., Suksamrarn A. (2003). Effects of elephant garlic volatile oil (*Allium ampeloprasum*) and T-2 Toxin on Murine Skin. Southeast Asian J. Trop. Med. Public Health.

[126] Nasir A.S. (2012). Hepatoprotective and some haematological parameters effect of *Allium ampeloprasum* against carbon tetrachloride induced liver toxicity in albino rats. Kufa J. Vet. Med. Sci..

[127] Badary O.A., Yassin N.A.Z., El-Shenawy S.M.A., EL-Moneem M.A., AL-Shafeiy H.M. (2013). Study of the effect of *Allium porrum* on hypertension induced in rats. Rev. Latinoam. Química.

[128] Rahimi-Madiseh M., Heidarian E., Kheiri S., Rafieian-Kopaei M. (2017). Effect of hydroalcoholic *Allium ampeloprasum* extract on oxidative stress, diabetes mellitus and dyslipidemia in alloxan-induced diabetic rats. Biomed. Pharmacother..

[129] Parvu A.E., Parvu M., Vlase L., Miclea P., Mot A.C., Silaghi-Dumitrescu R. (2014). Anti-inflammatory effects of *Allium schoenoprasum* L. leaves. J. Physiol. Pharmacol..

[130] Dobhal Y., Parcha V., Dhasmana D.C. (2014). Cardioprotective potential of *Allium humile* leaves extract. Orient. Pharm. Exp. Med..

[131] Dobhal Y., Parcha V., Dhasmana D.C. (2015). Effect of cardioactive principle of methanolic extract of *Allium humile* leaves on global ischaemic rat heart. Pharm. Biol. Eval..

[132] Rafieian-kopaei M., Keshvari M., Asgary S., Salimi M., Heidarian E. (2013). Potential role of a nutraceutical spice (*Allium hirtifolium*) in reduction of atherosclerotic plaques. J. HerbMed Pharmacol..

[133] Suzuki K.T., Tsuji Y., Ohta Y., Suzuki N. (2008). Preferential organ distribution of methylselenol source Se-methylselenocysteine relative to methylseleninic acid. Toxicol. Appl. Pharmacol..

[134] Ip C., Lisk D.J. (1995). Efficacy of cancer prevention by high-selenium garlic is primarily dependent on the action of Selenium. Carcinogenesis.

[135] El-Bayoumy K., Sinha R., Pinto J.T., Rivlin R.S. (2006). Cancer chemoprevention by garlic and garlic-containing Sulfur and Selenium compounds. J. Nutr..

[136] Imen A., Najjaa H., Neffati M. (2013). Influence of sulfur fertilization on S-containing, phenolic, and carbohydrate metabolites in rosy garlic (*Allium roseum* L.): A wild edible species in North Africa. Eur. Food Res. Technol..

[137] Zeng H., Combs G.F. (2008). Selenium as an anticancer nutrient: Roles in cell proliferation and tumor cell invasion. J. Nutr. Biochem..

[138] Ogra Y., Ishiwata K., Iwashita Y., Suzuki K.T. (2005). Simultaneous speciation of Selenium and Sulfur species in selenized odorless garlic (*Allium sativum* L. Shiro) and shallot (*Allium ascalonicum*) by HPLC-inductively coupled plasma-(octopole reaction system)-mass spectrometry and electrospray ionization-tand. J. Chromatogr. A.

[139] Dong Y., Lisk D., Block E., Ip C. (2001). Characterization of the biological activity of γ-glutamyl-Se—methylselenocysteine: A novel, naturally occurring anticancer agent from garlic. Cancer Res..

[140] Shah M., Kannamkumarath S.S., Wuilloud J.C.A., Wuilloud R.G., Caruso J.A. (2004). Identification and characterization of Selenium species in enriched green onion (*Allium fistulosum*) by HPLC-ICP-MS and ESI-ITMS. J. Anal. At. Spectrom..

[141] Tsuneyoshi T., Yoshida J., Sasaoka T. (2006). Hydroponic cultivation offers a practical means of producing Selenium enriched garlic. J. Nutr..

[142] Coolong T.W., Randle W.M. (2003). Sulfur and Nitrogen availability interact to affect the flavor biosynthetic pathway in onion. J. Am. Soc. Hortic. Sci..

[143] Forney C.F., Jordan M.A., Campbell-Palmer L., Fillmore S., McRae K., Best K. (2010). Sulfur fertilization affects onion quality and flavor chemistry during storage. Acta Hortic..

[144] Kumari K., Augusti K.T. (2002). Antidiabetic and antioxidant effects of S -methyl cysteine sulfoxide isolated from onions (*Allium cepa* Linn) as compared to standard drugs in alloxan diabetic rats. Indian J. Exp. Biol..

[145] Bloem E., Haneklaus S., Schnug E. (2005). Influence of Nitrogen and Sulfur fertilization on the Alliin content of onions and garlic. J. Plant Nutr..

[146] Bloem E., Haneklaus S., Schnug E. (2010). Influence of fertilizer practices on S-containing metabolites in garlic (*Allium sativum* L.) under field conditions. J. Agric. Food Chem..

[147] Nasim S.A., Dhir B., Samar F., Rashmi K., Mahmooduzzafar M.A. (2009). Sulphur treatment alters the therapeutic potency of alliin obtained from garlic leaf extract. Food Chem. Toxicol..

[148] Navarro-Alarcon M., Cabrera-Vique C. (2008). Selenium in food and the human body: A review. Sci. Total Environ..

[149] Fordyce F.M., Selinus O. (2013). Selenium deficiency and toxicity in the environment. Essentials of Medical Geology.

[150] De Temmerman L., Waegeneers N., Thiry C., Du Laing G., Tack F., Ruttens A. (2014). Selenium content of Belgian cultivated soils and its uptake by field crops and vegetables. Sci. Total Environ..

[151] Feng R., Wei C., Tu S. (2013). The roles of Selenium in protecting plants against abiotic stresses. Environ. Exp. Bot..

[152] Hurst R., Siyame E.W.P., Young S.D., Chilimba A.D.C., Joy E.J.M., Black C.R., Ander E.L., Watts M.J., Chilima B., Gondwe J. (2013). Soil-type influences human Selenium status and underlies widespread Selenium deficiency risks in Malawi. Sci. Rep..

[153] Hanousek O., Mason S., Santner J., Chowdhury M.M.A., Berger T.W., Prohaska T. (2016). Novel diffusive gradients in thin films technique to assess labile sulfate in soil. Anal. Bioanal. Chem..

[154] Anderson G.C., Peverill K.I., Brennan R.F. (2013). Soil potassium—Crop response calibration relationships and criteria for field crops grown in Australia. Crop Pasture Sci..

[155] Piotrowska-Długosz A., Siwik-Ziomek A., Długosz J., Gozdowski D. (2017). Spatio-temporal variability of soil Sulfur content and arylsulfatase activity at a conventionally managed arable field. Geoderma.

[156] Ahmed H.P., Schoenau J.J., King T., Kar G. (2017). Effects of seed-placed sulfur fertilizers on canola, wheat, and pea yield; Sulfur uptake; and soil sulfate concentrations over time in three prairie soils. J. Plant Nutr..

[157] Sarfaraz Q., Perveen S., Shahab Q., Muhammad D., Bashir S., Ahmed N., Ahmed S., Shahid-ul-islam M., Asghar I. (2014). Comparative effect of soil and foliar application of sulfur on maize. J. Agric. Vet. Sci..

[158] López-Gutiérrez M.L., Benavides-Mendoza A., Ortega-Ortíz H., Valdez-Aguilar L.A., Cabrera-De la Fuente M., Sandoval-Rangel A. (2015). Selenium and its effect on antioxidant status and mineral composition of lettuce. Rev. Mex. Ciencias Agricolas.

[159] Põldma P., Moor U., Tõnutare T., Herodes K., Rebane R. (2013). Selenium treatment under field conditions affects mineral nutrition, yield and antioxidant properties of bulb onion (*Allium cepa* L.). Acta Sci. Pol. Hortorum Cultus.

[160] Sánchez-Rodas D., Mellano F., Martínez F., Palencia P., Giráldez I., Morales E. (2016). Speciation analysis of Se-enriched strawberries (*Fragaria ananassa* Duch) cultivated on hydroponics by HPLC-TR-HG-AFS. Microchem. J..

[161] De los Santos-Vázquez M.E., Benavides-Mendoza A., Ruiz-Torres N.A., Cabrera-De la Fuente M., Morelos-Moreno A. (2016). Sodium selenite treatment of vegetable seeds and seedlings and the effect on antioxidant status. Emirates J. Food Agric..

[162] Chen C.C., Sung J.M. (2001). Priming bitter gourd seeds with Selenium solution enhances germinability and antioxidative responses under sub-optimal temperature. Physiol. Plant..

[163] Businelli D., D’Amato R., Onofri A., Tedeschini E., Tei F. (2015). Se-enrichment of cucumber (*Cucumis sativus* L.), lettuce (*Lactuca sativa* L.) and tomato (*Solanum lycopersicum* L. Karst) through fortification in pre-transplanting. Sci. Hortic..

[164] Orvis K.S., Goldman I.L. (1997). Relationship between antiplatelet activity and Sulfur fertility in hydroponic and field-grown onions (*Allium cepa*). HortScience.

[165] Zhang L., Hu B., Li W., Che R., Deng K., Li H., Yu F., Ling H., Li Y., Chu C. (2014). OsPT2, a phosphate transporter, is involved in the active uptake of selenite in rice. New Phytol..

[166] Li H.-F., McGrath S.P., Zhao F.-J. (2008). Selenium uptake, translocation and speciation in wheat supplied with selenate or selenite. New Phytol..

[167] Zhao X.Q., Mitani N., Yamaji N., Shen R.F., Ma J.F. (2010). Involvement of Silicon influx transporter OsNIP2;1 in selenite uptake in rice. Plant Physiol..

[168] Hopper J.L., Parker D.R. (1999). Plant availability of selenite and selenate as influenced by the competing ions phosphate and sulfate. Plant Soil.

[169] Roca-Perez L., Gil C., Cervera M.L., Gonzálvez A., Ramos-Miras J., Pons V., Bech J., Boluda R. (2010). Selenium and heavy metals content in some Mediterranean soils. J. Geochem. Explor..

[170] Zayed A., Lytle C.M., Terry N. (1998). Accumulation and volatilization of different chemical species of Selenium by plants. Planta.

[171] Zimmerman M.T., Bayse C.A., Ramoutar R.R., Brumaghim J.L. (2015). Sulfur and selenium antioxidants: Challenging radical scavenging mechanisms and developing structure-activity relationships based on metal binding. J. Inorg. Biochem..

[172] Anjum N.A., Gill R., Kaushik M., Hasanuzzaman M., Pereira E., Ahmad I., Tuteja N., Gill S.S. (2015). ATP-sulfurylase, Sulfur-compounds, and plant stress tolerance. Front. Plant Sci..

[173] Sharma N., Kumar A., Prakash R., Prakash N.T. (2007). Selenium accumulation and Se-induced anti-oxidant activity in *Allium cepa*. Environ. Inform. Arch..

[174] Bystrická J., Kavalcová P., Musilová J., Tomáš J., Tóth T., Orsák M. (2015). Selenium and Its influence on the content of polyphenol compounds in onion (*Allium cepa* L.). J. Microbiol. Biotechnol. Food Sci..

[175] Põldma P., Tõnutare T., Viitak A., Luik A., Moor U. (2011). Effect of Selenium treatment on mineral nutrition, bulb size, and antioxidant properties of garlic (*Allium sativum* L.). J. Agric. Food Chem..

[176] Cheng B., Lian H.F., Liu Y.Y., Yu X.H., Sun Y.L., Sun X.D., Shi Q.H., Liu S.Q. (2016). Effects of Selenium and Sulfur on antioxidants and physiological parameters of garlic plants during senescence. J. Integr. Agric..

[177] Zhao J., Hu Y., Gao Y., Li Y., Li B., Dong Y., Chai Z. (2013). Mercury modulates Selenium activity via altering its accumulation and speciation in garlic (*Allium sativum*). Metallomics.

[178] Yadav S., Gupta S., Prakash R., Spallholz J., Prakash N.T. (2007). Selenium uptake by *Allium cepa* grown in Se-spiked soils. Am. J. Agric. Environ. Sci..

[179] Afton S.E., Caruso J.A. (2009). The effect of Se antagonism on the metabolic fate of Hg in *Allium fistulosum*. J. Anal. At. Spectrom..

[180] Zhao J., Gao Y., Li Y.-F., Hu Y., Peng X., Dong Y., Li B., Chen C., Chai Z. (2013). Selenium inhibits the phytotoxicity of Mercury in garlic (*Allium sativum*). Environ. Res..

[181] Kopsell D.A., Randle W.M. (1997). Selenate concentration affects Selenium and Sulfur uptake and accumulation by “Granex 33” onions. J. Am. Soc. Hortic. Sci..

[182] Kápolna E., Shah M., Caruso J.A., Fodor P. (2007). Selenium speciation studies in Se-enriched chives (*Allium schoenoprasum*) by HPLC-ICP-MS. Food Chem..

[183] Randle W.M., Block E., Littlejohn M.H., Putman D., Bussard M.L. (1994). Onion (*Allium cepa* L.) thiosulfinates respond to increasing sulfur fertility. J. Agric. Food Chem..

[184] Liu S., He H., Feng G., Chen Q. (2009). Effect of Nitrogen and Sulfur interaction on growth and pungency of different pseudostem types of Chinese spring onion (*Allium fistulosum* L.). Sci. Hortic..

[185] Mishu H.M., Ahmed F., Rafii M.Y., Golam F., Latif M.A. (2013). Effect of Sulphur on growth, yield and yield attributes in onion (*Allium cepa* L.). Aust. J. Crop Sci..

[186] Nasreen S., Imamul Haq S., Hossain A. (2003). Sulphur effects on growth responses and yield of onion. Asian J. Plant Sci..

[187] Randle W.M. (1992). Onion germplasm interacts with Sulfur fertility for plant sulfur utilization and bulb pungency. Euphytica.

[188] Randle W.M., Bussard M.L. (1993). Pungency and sugars of short-day onions as affected by Sulfur nutrition. J. Am. Soc. Hortic. Sci..

[189] McCallum J., Porter N., Searle B., Shaw M., Bettjeman B., McManus M. (2005). Sulfur and Nitrogen fertility affects flavour of field-grown onions. Plant Soil.

[190] Bolandnazar S., Mollavali M., Tabatabaei S.J. (2012). Influence of NH_4_NO_3_ and K_2_SO_4_ on qualitative characteristics of onion. Sci. Hortic..

[191] Guo T., Zhang J., Christie P., Li X. (2007). Pungency of Spring Onion as affected by inoculation with arbuscular mycorrhizal fungi and Sulfur supply. J. Plant Nutr..

[192] Hamilton B.K., Pike L.M., Yoo K.S. (1997). Clonal variations of pungency, sugar content, and bulb weight of onions due to Sulphur nutrition. Sci. Hortic..

[193] Coolong T.W., Kopsell D.A., Kopsell D.E., Randle W.M. (2004). Nitrogen and Sulfur influence nutrient usage and accumulation in onion. J. Plant Nutr..

[194] Youssif B.D., Hosna M.A.F., Mervat A.A.T. (2015). Effect of Sulphur and Sulphur oxidizing bacteria on growth and production of garlic (*Allium sativum*, L.) under saline conditions. Middle East J. Agric. Res..

[195] Kápolna E., Fodor P. (2006). Speciation analysis of Selenium enriched green onions (*Allium fistulosum*) by HPLC-ICP-MS. Microchem. J..

[196] Prakash N.T., Sharma N., Prakash R., Nathaniel T.N., Acharya R., Reddy A.V.R. (2010). Selenium fortification and pro/anti oxidant responses in *Allium cepa* (onion) cultivated in Se supplemented soils. Exp. Agric..

[197] Guo T., Zhang J., Christie P., Li X. (2006). Influence of Nitrogen and Sulfur fertilizers and inoculation with arbuscular mycorrhizal fungi on yield and pungency of spring onion. J. Plant Nutr..

[198] Hamilton B.K., Yoo K.S., Pike L.M. (1998). Changes in pungency of onions by soil type, Sulphur nutrition and bulb maturity. Sci. Hortic..

[199] Nasreen S., Haque M.M., Hossain M.A., Farid A.T.M. (2007). Nutrient uptake and yield of onion as influenced by Nitrogen and Sulphur fertilization. Bangladesh J. Agric. Res..

[200] De Souza L.F.G., Filho A.B.C., de Túlio F.A., Nowaki R.H.D. (2015). Effect of Sulphur dose on the productivity and quality of onions. Aust. J. Crop Sci..

[201] Díaz-Pérez J.C., Bautista J., Bateman A., Gunawati G., Riner C. (2016). Sweet onion (*Allium cepa*) plant growth and bulb yield and quality as affected by Potassium and Sulfur fertilization rates. HortScience.

[202] Lee E.J., Yoo K.S., Jifon J., Patil B.S. (2009). Application of extra Sulfur to high-Sulfur soils does not increase pungency and related compounds in shortday onions. Sci. Hortic..

[203] Randle W.M., Bussard M.L., Warnock D.F. (1993). Ontogeny and Sulfur fertility affect leaf Sulfur in short-day onions. J. Am. Soc. Hortic. Sci..

[204] Randle W.M., Lancaster J.E., Shaw M.L., Sutton K.H., Hay R.L., Bussard M.L. (1995). Quantifying onion flavor compounds responding to Sulfur fertility-Sulfur increases levels of Alk(en)yl Cysteine Sulfoxides and biosynthetic intermediates. J. Am. Soc. Hortic. Sci..

